# Manipulation of the Host Cell Membrane during *Plasmodium* Liver Stage Egress

**DOI:** 10.1128/mBio.00139-17

**Published:** 2017-04-11

**Authors:** Paul-Christian Burda, Reto Caldelari, Volker T. Heussler

**Affiliations:** Institute of Cell Biology, University of Bern, Bern, Switzerland; NIAID/NIH

## Abstract

A crucial step in the life cycle of *Plasmodium* parasites is the transition from the liver stage to the blood stage. Hepatocyte-derived merozoites reach the blood vessels of the liver inside host cell-derived vesicles called merosomes. The molecular basis of merosome formation is only partially understood. Here we show that *Plasmodium berghei* liver stage merozoites, upon rupture of the parasitophorous vacuole membrane, destabilize the host cell membrane (HCM) and induce separation of the host cell actin cytoskeleton from the HCM. At the same time, the phospholipid and protein composition of the HCM appears to be substantially altered. This includes the loss of a phosphatidylinositol 4,5-bisphosphate (PIP_2_) reporter and the PIP_2_-dependent actin-plasma membrane linker ezrin from the HCM. Furthermore, transmembrane domain-containing proteins and palmitoylated and myristoylated proteins, as well as glycosylphosphatidylinositol-anchored proteins, lose their HCM localization. Collectively, these findings provide an explanation of HCM destabilization during *Plasmodium* liver stage egress and thereby contribute to our understanding of the molecular mechanisms that lead to merosome formation.

## IMPORTANCE

Egress from host cells is an essential process for intracellular pathogens, allowing successful infection of other cells and thereby spreading the infection. Here we describe the molecular details of a novel egress strategy of *Plasmodium* parasites infecting hepatocytes. We show that toward the end of the liver stage, parasites induce a breakdown of the host cell actin cytoskeleton, leading to destabilization of the host cell plasma membrane. This, in turn, results in the formation of membrane vesicles (merosomes), in which parasites can safely migrate from liver tissue to the bloodstream to infect red blood cells and start the pathogenic phase of malaria.

## INTRODUCTION

Malaria is caused by parasites of the genus *Plasmodium* and remains one of the most important infectious diseases worldwide, with more than 200 million people infected and about 430,000 deaths per year (1). Symptoms arise from multiplication of parasites within the blood, while the preceding liver stage of infection is asymptomatic. Central to the initiation of blood stage development is the transition of parasites from the liver to the blood. This occurs via the formation of hepatocyte-derived vesicles termed merosomes, in which parasites, unrecognized by the host immune system, are transported to the blood circulation (2).

Merosome formation is associated with a unique kind of host cell death that shares some features of typical apoptosis, such as mitochondrial disintegration and nuclear condensation, but lacks others, such as caspase activation. Importantly, host cell membrane (HCM) integrity is preserved, since by their active uptake of calcium, parasites prevent exposure of phosphatidylserine (PS) to the outer leaflet of the merosomal membrane, which normally acts as a typical “eat me” signal for phagocytic cells during apoptosis. During merosome formation, host hepatocytes undergo remarkable morphological changes. While *in vivo* very flexible merosomes are formed that squeeze through the liver endothelium, *in vitro* infected host cells lose contact with the culture dish and can be found as rounded-up detached cells (2).

Since the cytoskeleton is a key regulator of cell adhesion and deformability, it is an attractive hypothesis that parasites actively change the host cytoskeleton during merosome formation. Interestingly, a modification of the host cell cytoskeleton has already been demonstrated to occur during the release of *Plasmodium falciparum* blood stage parasites. Blood stage egress was shown to depend on host cell calpain-1, which degrades erythrocyte cytoskeletal proteins during parasite release (3). In addition, a proteomic study provided evidence that *Plasmodium* blood stage parasites actively weaken the HCM in the last 15 to 20 h before egress by removing cytoskeletal proteins such as adducins in order to prepare the infected red blood cell (RBC) for rupture (4). Furthermore, it could recently be shown that in the final minutes of egress, the blood cell membrane abruptly loses its structural rigidity and collapses around parasites, suggesting a breakdown of the RBC cytoskeleton (5). In contrast to *P. falciparum* blood stages, the fate of the host cell cytoskeleton during *Plasmodium berghei* hepatocyte infection and how the HCM is destabilized during merosome formation have so far not been investigated.

In this study, we characterize the parasite-induced changes to the host cell cytoskeleton and plasma membrane during liver stage egress by live-cell time-lapse imaging with fluorescent reporter proteins. We show that parasites destroy host cell actin-plasma membrane linkage during merosome formation and that, at the same time, the phospholipid and protein composition of the HCM appears to be substantially altered.

## RESULTS

### *Plasmodium* parasites destroy host cell actin-plasma membrane linkage during egress from host hepatocytes.

**FIG 1:**
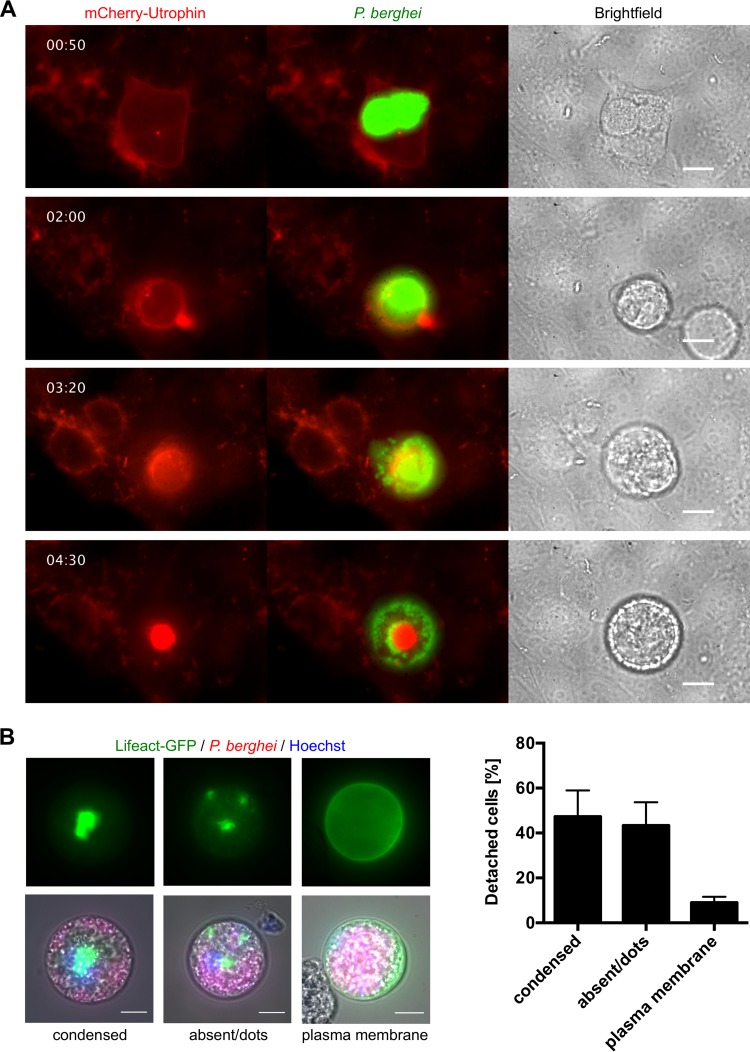
Host cell actin-plasma membrane linkage is lost during *Plasmodium* egress from host hepatocytes. (A) HeLa cells were transfected with a construct encoding mCherry-utrophin (red) and infected with GFP-expressing parasites (green). Shown are stills of a live-cell imaging movie that was started at 62 hpi. (B) Representative images of the different types of actin localization (condensed, absent or dots, and plasma membrane associated) in detached cells derived from primary Lifeact hepatocytes. Primary hepatocytes were isolated from a Lifeact mouse and infected with mCherry-expressing parasites (red). At 65 hpi, detached cells were harvested and analyzed by live-cell microscopy. Nuclei were visualized with Hoechst 33342 (blue). The actin cytoskeleton is shown in green. The relative percentage of each type of actin localization is shown as the mean and the standard error of the mean on the right and was quantified in three independent experiments in which a total of 189 detached cells were analyzed. All scale bars, 10 µm. See also Movies S1 and S2.

As the cortical actin cytoskeleton plays a major role in the regulation of cell adhesion and cell deformability (reviewed in reference 6), we wondered whether the actin cytoskeleton is altered during the remarkable changes the host cell undergoes after rupture of the parasitophorous vacuole membrane (PVM) and subsequent merosome formation. We therefore expressed an mCherry-tagged version of the filamentous actin-binding protein utrophin (7) in HeLa cells, infected them with green fluorescent protein (GFP)-expressing *P. berghei* parasites, and monitored their development by live-cell time-lapse microscopy. Interestingly, after PVM rupture, the cortical actin cytoskeleton detached from the host cell plasma membrane and the plasma membrane formed large blebs (Fig. 1A; see Movie S1 in the supplemental material), which were sometimes larger than the diameter of the cell itself. Subsequently, the actin cytoskeleton completely separated from the HCM and collapsed into the center of the cell. This resulted in rounding up of the detached cell, termination of blebbing, and free distribution of merozoites in the host cell cytoplasm (Fig. 1A; see Movie S1).

10.1128/mBio.00139-17.1 MOVIE S1  *Plasmodium* liver stages induce host cell blebbing and dissociation of the actin cytoskeleton from the HCM during egress (related to Fig. 1). HeLa cells expressing mCherry-utrophin (red) were infected with GFP-expressing parasites (green). Parasite development was monitored by epifluorescence live-cell time-lapse microscopy, and imaging was started at 62 hpi. The movie was acquired with a 10-min time interval between frames and is shown at four frames per second. Hours and minutes from the start of the movie are displayed. Scale bar, 10 μm. Download MOVIE S1, AVI file, 2.6 MB. Copyright © 2017 Burda et al. 2017 Burda et al. This content is distributed under the terms of the Creative Commons Attribution 4.0 International license.

To exclude the possibility that this phenomenon is an artifact of the HeLa cell line, we next infected primary hepatocytes and investigated their actin dynamics during *Plasmodium*-induced cell detachment. For this, primary hepatocytes were isolated from Lifeact-GFP mice that ubiquitously express the 17-amino-acid Lifeact peptide fused to GFP, which specifically labels filamentous actin without adverse effects on actin dynamics (8). Importantly, in primary hepatocytes, the actin cytoskeleton also lost its connection to the HCM during liver stage egress (see Movie S2). Quantification of the distribution of actin in detached primary hepatocytes revealed that in the majority of these cells, the actin cytoskeleton had separated from the plasma membrane and was found either as a condensed structure or small dots or was completely absent (Fig. 1B), further supporting our live-cell imaging data.

10.1128/mBio.00139-17.2 MOVIE S2  Host cell actin modulation occurs in primary Lifeact hepatocytes (related to Fig. 1). Primary hepatocytes derived from a Lifeact mouse (green) were infected with mCherry-expressing parasites (red). Parasite development was monitored by confocal live-cell time-lapse microscopy, and imaging was started at 57 hpi. The movie was acquired with a 10-min time interval between frames and is shown at four frames per second. Hours and minutes from the start of the movie are displayed. Scale bar, 10 μm. Download MOVIE S2, AVI file, 1.2 MB. Copyright © 2017 Burda et al. 2017 Burda et al. This content is distributed under the terms of the Creative Commons Attribution 4.0 International license.

### E64 treatment interferes with manipulation of the host cell actin cytoskeleton.

**FIG 2:**
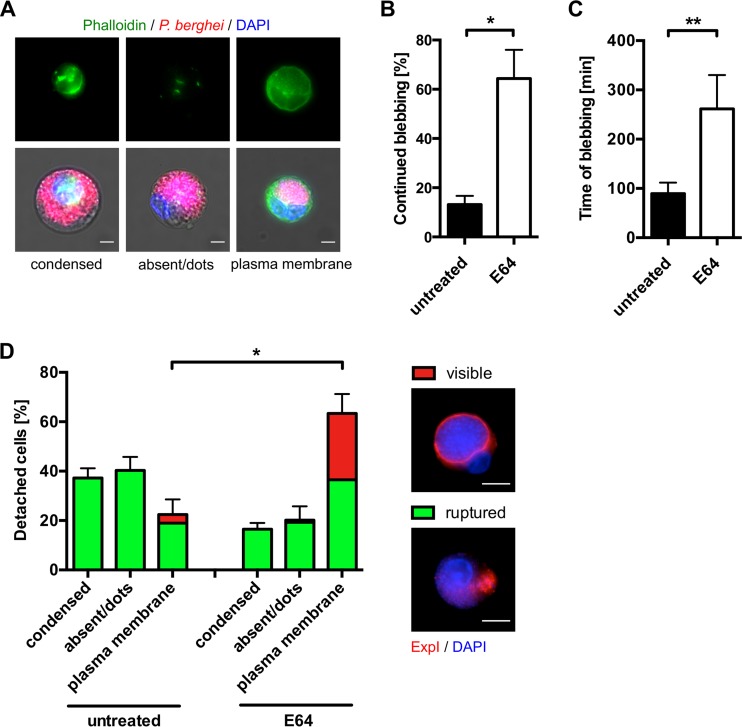
Treatment with the general cysteine protease inhibitor E64 interferes with modulation of the host cell actin cytoskeleton. (A) Different types of actin localization (condensed, absent or dots, and plasma membrane associated) in phalloidin-stained detached cells. HeLa cells were infected with mCherry-expressing parasites (red), and at 65 hpi, detached cells were harvested, fixed, and stained with phalloidin (green). Nuclei were visualized with DAPI (blue). (B, C) E64 treatment prevents termination of *Plasmodium*-induced blebbing. HeLa cells were infected with mCherry-expressing parasites and at 55 hpi treated with 10 µg/ml E64 or left untreated. *Plasmodium*-induced blebbing was analyzed by live-cell time-lapse imaging starting at 56 hpi for a period of 12 h. Thereby, only those parasites were analyzed that started blebbing within the first 6 h of imaging. The percentage of infected cells that continued blebbing and did not terminate the blebbing process within the imaging time is shown in panel B. The average blebbing time of infected cells that terminated the blebbing process is displayed in panel C. Shown are the mean and the standard error of the mean of three independent imaging experiments in which a total of 37 egress events of untreated and 25 egress events of E64-treated parasites were observed. Eleven untreated and four E64-treated infected cells did not show blebbing and were not included in the analysis. For statistical analysis, an unpaired two-tailed *t* test was performed. Statistically significant differences are indicated by asterisks (*, *P* < 0.05; **, *P* < 0.01). See also Movie S3. (D) Quantification of actin localization after E64-mediated inhibition of complete PVM rupture. HeLa cells were infected with parasites expressing the PVM marker protein ExpI fused to mCherry. At 56 hpi, 10 µg/ml E64 was added or cells were left untreated. At 65 hpi, detached cells were harvested, fixed, and stained with phalloidin. The percentages of the different types of actin localization were quantified in three independent experiments in which 221 untreated and 232 E64-treated detached cells were analyzed in total. Shown are the mean and the standard error of the mean. Cells with a ruptured PVM are indicated in green, while cells with a visible PVM are indicated in red. Representative detached cells with a visible and ruptured PVM are shown on the right, where the PVM marker ExpI is displayed in red and DAPI-stained nuclei are shown in blue. For statistical analysis, an unpaired two-tailed *t* test was performed. Statistically significant differences are indicated by asterisks (*, *P* < 0.05). All scale bars, 10 μm.

To exclude the possibility that the apparent loss of actin-plasma membrane linkage during liver stage egress is an artifact of the mCherry-utrophin or Lifeact-GFP protein, we harvested detached cells derived from nontransfected HeLa cells, fixed them, and stained their actin cytoskeleton with phalloidin. Similar to our results illustrated in Fig. 1, phalloidin staining also revealed that the actin cytoskeleton had lost its connection to the plasma membrane in the majority of detached cells (Fig. 2A and D), indicating that the observed effects are not an artifact of the actin-binding proteins used for visualization (Fig. 1).

Next, we analyzed the role of PVM rupture in the modulation of the host actin cytoskeleton and made use of the general cysteine protease inhibitor E64, which has been shown to exert a different effect on blood and liver stage egress. E64 treatment of blood stage parasites allows PVM rupture but selectively prevents erythrocyte membrane rupture (5, 9). In contrast, E64 treatment of late liver stage parasites appears to prevent complete PVM rupture. Some cells still detach, but in most of these detached cells, the PVM is incompletely ruptured and still visible, meaning that merozoites are not liberated into the host cell cytoplasm (2).

Here, we first treated infected cells shortly before PVM rupture with E64 or left them untreated and monitored parasite development by live-cell imaging. Interestingly, in E64-treated infected cells, we frequently observed cells that continued blebbing for a prolonged period of time and in which the host actin cytoskeleton remained associated with the plasma membrane (see Movie S3). Quantification of this phenomenon revealed that significantly more E64-treated infected cells than untreated cells continued blebbing during the 12 h of imaging time (Fig. 2B). Furthermore, when we looked at the proportion of cells that still did terminate the blebbing process, the blebbing time was significantly longer in E64-treated cells than in untreated cells (Fig. 2C).

10.1128/mBio.00139-17.3 MOVIE S3  E64 treatment leads to unterminated blebbing and prevents actin modulation (related to Fig. 2). HeLa cells expressing GFP-utrophin (green) were infected with mCherry-expressing parasites (red) and treated with 10 μg/ml E64 at 55 hpi. Parasite development was monitored by epifluorescence live-cell time-lapse microscopy, and imaging was started at 56 hpi. The movie was acquired with a 10-min time interval between frames and is shown at four frames per second. Hours and minutes from the start of the movie are displayed. Scale bar, 10 μm. Download MOVIE S3, AVI file, 2.3 MB. Copyright © 2017 Burda et al. 2017 Burda et al. This content is distributed under the terms of the Creative Commons Attribution 4.0 International license.

To investigate this further, we infected HeLa cells with parasites expressing a fluorescently tagged version of the PVM marker protein ExpI (10) and treated them with E64 or left them untreated. When detached cells from E64-treated cultures were harvested and stained with phalloidin, the percentage of cells in which the actin cytoskeleton was still associated with the plasma membrane was statistically significantly greater than that of untreated cells and in about half of these cells, the PVM was still visible (Fig. 2D). Importantly, we hardly ever observed a detached cell with a visible PVM in which the actin cytoskeleton had lost its association with the plasma membrane. Together, these findings indicate that complete rupture of the PVM and release of merozoites into the host cell cytoplasm are necessary for detachment of the host actin cytoskeleton from the plasma membrane. However, it is important to mention that it is also possible that E64 inhibits one or more cysteine proteases directly or indirectly responsible for severing the linkages between the actin cytoskeleton and the plasma membrane, which might be independent of its inhibitory role for liver stage PVM rupture.

### Shear forces are necessary for merosome formation.

**FIG 3:**
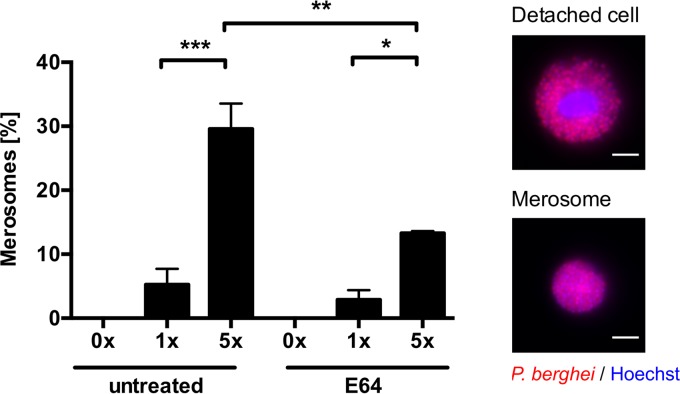
*In vitro* merosome formation is dependent on shear forces and is sensitive to E64. HeLa cells were infected with mCherry-expressing parasites, and at 55 hpi, cells were treated with 10 µg/ml E64 or left untreated. At 65 hpi, the detached-cell-containing cell culture supernatant was either directly analyzed without pipetting (0×) or pipetted up and down one or five times (1×, 5×) before analysis. Detached cells (containing a host cell nucleus) and merosomes (lacking a host cell nucleus) were discriminated by staining with Hoechst 33342. Representative images of detached cells and merosomes are shown on the right, where the parasite cytoplasm is displayed in red and nuclei are in blue. The relative percentage of merosome formation is shown as the mean and the standard error of the mean of three independent experiments in which a total of 1,134 untreated and 811 E64-treated detached cells and merosomes were analyzed. For statistical analysis, a one-way ANOVA, followed by a Holm-Sidak multiple-comparison test, was performed. Statistically significant differences are indicated by asterisks (*, *P* < 0.05; **, *P* < 0.01; ***, *P* < 0.001). All scale bars, 10 µm.

*In vivo*, quantitative intravital microscopy revealed merosome formation (2), and *in vitro*, merosomes can be detected when the supernatant of infected cells is analyzed. However, *in vitro*, the actual process of merosome formation and separation from the mother cell has never been recorded, despite considerable effort. Since for counting of detached cells and merosomes, the culture supernatant of infected cells is normally transferred into a fresh culture plate, we reasoned that shear forces during pipetting might be required to separate merosomes from mother cells. To test this hypothesis, we either did not transfer the culture supernatant of infected host cells at all or pipetted it up and down one or five times before analysis. In line with our hypothesis, we did not see any merosome formation, when we directly analyzed the culture supernatant without any pipetting. In contrast to this, merosome formation was visible when detached cells were subjected to further shear forces exerted by pipetting (Fig. 3). Interestingly, treatment of infected cells with E64 before detachment significantly decreased merosome formation *in vitro*. These observations, in combination with the finding that E64 treatment prevents detachment of the actin cytoskeleton from the HCM (Fig. 2D), suggest that both modulation of the actin cytoskeleton and shear forces are necessary for merosome formation to occur.

### Phospholipid reporters are lost from the HCM upon PVM rupture.

**FIG 4:**
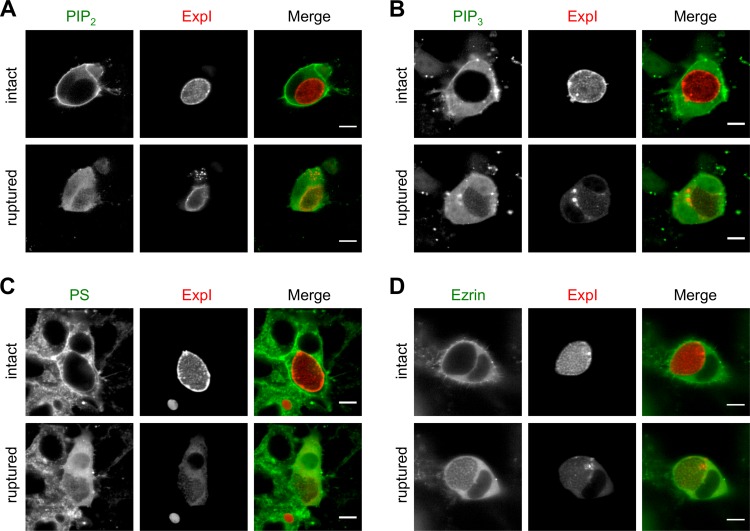
The phospholipid composition of the HCM appears to be altered during liver stage egress. HeLa cells were transfected with constructs encoding the PH domain of phospholipase C-δ1 fused to GFP to visualize PIP_2_ (A), the PH domain of AKT serine/threonine kinase 1 fused to GFP to visualize PIP_3_ (B), the lactadherin-C2 domain fused to GFP to visualize PS (C), and an ezrin-GFP fusion protein (D). Transfected cells were subsequently infected with parasites expressing mCherry fused to the PVM marker protein ExpI (red), and parasite development was monitored by live-cell time-lapse microscopy. The localization of the phospholipid reporters and ezrin (green) in infected cells with intact PVM and in the same cells with a ruptured PVM is shown. Results are representative of 26 (PIP_2_), 15 (PIP_3_), 19 (PS), and 20 (ezrin) analyzed egress events in two or three independent imaging experiments. All scale bars, 10 µm. See also Movies S4 and S5.

We next aimed to elucidate the molecular mechanisms underlying the observed dissociation of the actin cytoskeleton from the plasma membrane. One of the main factors in anchoring of the cortical actin cytoskeleton to the plasma membrane is phosphatidylinositol 4,5-bisphosphate (PIP_2_), which is found primarily in the plasma membrane. Here it promotes actin-plasma membrane linkage by binding and activating proteins such as the ERM proteins (ezrin, radixin, and moesin) that link the actin cytoskeleton to transmembrane proteins in the plasma membrane (reviewed in references 6 and 11). To investigate whether host cell PIP_2_ is modulated after PVM breakdown, we transfected HeLa cells to express the PH domain of phospholipase C-δ1 fused to GFP, which is commonly used to visualize PIP_2_ in the plasma membrane (12). Remarkably, upon rupture of the PVM, the PIP_2_ reporter was rapidly lost from the plasma membrane before or during host cell detachment (Fig. 4A; see Movie S4). To analyze whether other host cell phospholipids are also modulated upon PVM rupture, we expressed the PH domain of AKT serine/threonine kinase 1 and the lactadherin-C2 domain both fused to GFP in *P. berghei*-infected host cells. These constructs allow visualization of phosphatidylinositol 3,4,5-trisphosphate (PIP_3_) and PS, respectively (13, 14), and were, interestingly, similarly lost from the HCM upon PVM rupture (Fig. 4B and C). Together, these data suggest a general manipulation of the HCM phospholipid content by *Plasmodium* late liver stage parasites during egress, although we currently cannot completely exclude the possibility that loss of the phospholipid reporters from the HCM is due to unspecific effects such as proteolytic activity. However, the experiments described below and the fact that GFP fluorescence was not lost argue against unspecific proteolytic activity.

10.1128/mBio.00139-17.4 MOVIE S4  A fluorescent PIP_2_ reporter is lost from the HCM upon PVM rupture (related to Fig. 4). HeLa cells expressing the PH domain of phospholipase C-δ1 fused to GFP to visualize PIP_2_ (green) were infected with parasites expressing mCherry fused to the PVM marker protein ExpI (red). Parasite development was monitored by confocal live-cell time-lapse microscopy, and imaging was started at 56 hpi. The movie was acquired with a 10-min time interval between frames and is shown at four frames per second. Hours and minutes from the start of the movie are displayed. Scale bar, 10 μm. Download MOVIE S4, AVI file, 0.8 MB. Copyright © 2017 Burda et al. 2017 Burda et al. This content is distributed under the terms of the Creative Commons Attribution 4.0 International license.

As one of the key functions of PIP_2_ is to bind and activate several actin-binding proteins that lead to actin-plasma membrane linkage, we wondered whether downstream effectors of PIP_2_ are also affected by the apparent loss of host PIP_2_ from the HCM after PVM rupture. We therefore analyzed, during *Plasmodium*-induced cell detachment, the localization of a GFP fusion protein of the membrane-associated protein ezrin, which shows a localization very similar to that of PIP_2_ under normal circumstances (15). As expected, ezrin-GFP was also lost from the host cell plasma membrane upon rupture of the PVM and was only seen as a diffuse signal within detached cells (Fig. 4D; see Movie S5). This suggests that because of the apparent PIP_2_ loss, PIP_2_-dependent actin-plasma membrane linker proteins also lose their plasma membrane localization during liver stage egress. Remarkably, a constitutively active mutant version of ezrin (16) differing only in one amino acid (T567D) did not influence *Plasmodium*-induced cell detachment and showed a condensed and not diffuse signal in detached cells (see Movie S6). The different localization of wild-type and mutant ezrin proteins upon PVM rupture thus makes it unlikely that the observed loss of ezrin-GFP from the HCM is only due to unspecific proteolytic degradation of the fluorescent reporter.

10.1128/mBio.00139-17.5 MOVIE S5  Ezrin is lost from the HCM upon PVM rupture (related to Fig. 4). HeLa cells expressing ezrin-GFP (green) were infected with parasites expressing mCherry fused to the PVM marker protein ExpI (red). Parasite development was monitored by confocal live-cell time-lapse microscopy, and imaging was started at 55 hpi. The movie was acquired with a 10-min time interval between frames and is shown at four frames per second. Hours and minutes from the start of the movie are displayed. Scale bar, 10 μm. Download MOVIE S5, AVI file, 0.9 MB. Copyright © 2017 Burda et al. 2017 Burda et al. This content is distributed under the terms of the Creative Commons Attribution 4.0 International license.

10.1128/mBio.00139-17.6 MOVIE S6  A constitutively active mutant form of ezrin forms a condensed structure within detached cells (related to Fig. 4). HeLa cells expressing ezrin(T567D)-GFP (green) were infected with parasites expressing mCherry fused to the PVM marker protein ExpI (red). Parasite development was monitored by epifluorescence live-cell time-lapse microscopy, and imaging was started at 55 hpi. The movie was acquired with a 10-min time interval between frames and is shown at four frames per second. Hours and minutes from the start of the movie are displayed. Results are representative of 10 analyzed egress events. Scale bar, 10 μm. Download MOVIE S6, AVI file, 1.2 MB. Copyright © 2017 Burda et al. 2017 Burda et al. This content is distributed under the terms of the Creative Commons Attribution 4.0 International license.

### Membrane proteins are cleared from the HCM during liver stage egress.

**FIG 5:**
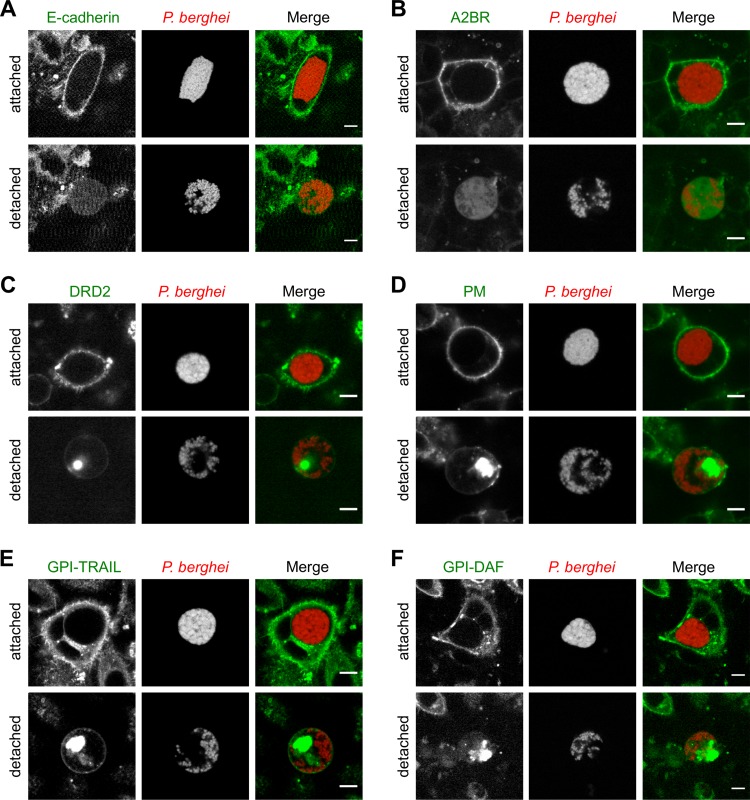
Membrane proteins are lost from the HCM during liver stage egress. HeLa cells were transfected with constructs encoding a GFP fusion of the single-pass transmembrane protein E-cadherin (A), a YFP fusion protein of the seven-transmembrane domain-containing A2BR (B), a GFP fusion protein of the seven-transmembrane domain-containing DRD2 (C), a GFP to which a conserved myristoylation-and-palmitoylation sequence from Lyn was added to the N-terminal end (PM) (D), GFP fusion proteins containing the GPI anchor sequence of either TRAIL-R3 (GPI-TRAIL) or decay-accelerating factor (GPI-DAF) at the C-terminal end (E, F). Transfected cells were subsequently infected with parasites expressing mCherry (red), and parasite development was monitored by live-cell time-lapse microscopy. The localization of proteins (green) in attached cells and in the same cells after cell detachment is shown. Results are representative of 25 (E-cadherin), 48 (A2BR), 18 (DRD2), 27 (PM), 34 (GPI-TRAIL), and 15 (GPI-DAF) analyzed egress events in two to four independent imaging experiments. All scale bars, 10 µm. See also Movies S7 and S8.

PIP_2_-dependent actin-plasma membrane linker proteins connect the actin cytoskeleton to membrane proteins in the HCM. We therefore determined the fate of these proteins during *Plasmodium*-induced cell detachment and first investigated the single-pass transmembrane protein E-cadherin, a protein central to cell-to-cell adhesion (17). Interestingly, E-cadherin–GFP lost its membrane localization during cell detachment and showed weak cytoplasmic staining in detached cells (Fig. 5A; see Movie S7). To also analyze how proteins containing multiple transmembrane domains, which are therefore firmly integrated in the HCM, behave upon PVM rupture, we expressed in infected HeLa cells yellow fluorescent protein (YFP)/GFP fusion proteins of the seven-transmembrane domain-containing proteins adenosine 2B receptor (A2BR) (18) and dopamine receptor D2 (DRD2) (19). Both proteins lost their membrane localization and were found either in the cytoplasm or as a condensed structure within detached cells (Fig. 5B and C).

10.1128/mBio.00139-17.7 MOVIE S7  E-cadherin is lost from the HCM during *Plasmodium*-induced cell detachment (related to Fig. 5). HeLa cells expressing E-cadherin–GFP (green) were infected with mCherry-expressing parasites (red). Parasite development was monitored by confocal live-cell time-lapse microscopy, and imaging was started at 55 hpi. The movie was acquired with a 10-min time interval between frames and is shown at four frames per second. Hours and minutes from the start of the movie are displayed. Scale bar, 10 μm. Download MOVIE S7, AVI file, 2.9 MB. Copyright © 2017 Burda et al. 2017 Burda et al. This content is distributed under the terms of the Creative Commons Attribution 4.0 International license.

Proteins can also be bound to the plasma membrane by palmitoylation and myristoylation or by addition of a glycosylphosphatidylinositol (GPI) anchor. To examine the fate of such proteins after PVM rupture, on the one hand, we expressed palmitoylated and myristoylated GFP (PM) in HeLa cells, which was generated by adding a conserved palmitoylation-and-myristoylation sequence from Lyn to the N terminus of GFP (20). On the other hand, we expressed in infected host cells GFP fusion proteins containing the GPI anchor sequences of either TRAIL-R3 or decay-accelerating factor at their C termini (GPI-TRAIL, GPI-DAF) (21). Remarkably, all of these GFP fusion proteins lost most of their membrane localization and formed a condensed or vesicular structure in detached cells (Fig. 5D to F; see Movie S8), suggesting that membrane proteins, independent of the presence of a transmembrane domain, are cleared from the HCM during *Plasmodium* liver stage egress.

10.1128/mBio.00139-17.8 MOVIE S8  Myristoylated and palmitoylated GFP (PM-GFP) forms a condensed structure within detached cells (related to Fig. 5). HeLa cells expressing PM-GFP (green) were infected with mCherry-expressing parasites (red). Parasite development was monitored by confocal live-cell time-lapse microscopy, and imaging was started at 56 hpi. The movie was acquired with a 10-min time interval between frames and is shown at four frames per second. Hours and minutes from the start of the movie are displayed. Scale bar, 10 μm. Download MOVIE S8, AVI file, 0.5 MB. Copyright © 2017 Burda et al. 2017 Burda et al. This content is distributed under the terms of the Creative Commons Attribution 4.0 International license.

## DISCUSSION

Leaving the liver within host cell-derived merosomes is a very effective strategy for *Plasmodium* parasites to enter the blood circulation without being recognized by the host immune system (2). To squeeze through very narrow gaps in the endothelium, the merosome membrane must be extremely flexible. Another prerequisite of merosome formation is that the infected cell detaches from neighboring cells. To achieve both, cell-cell connections and the host cell cytoskeleton must be modulated.

**FIG 6:**
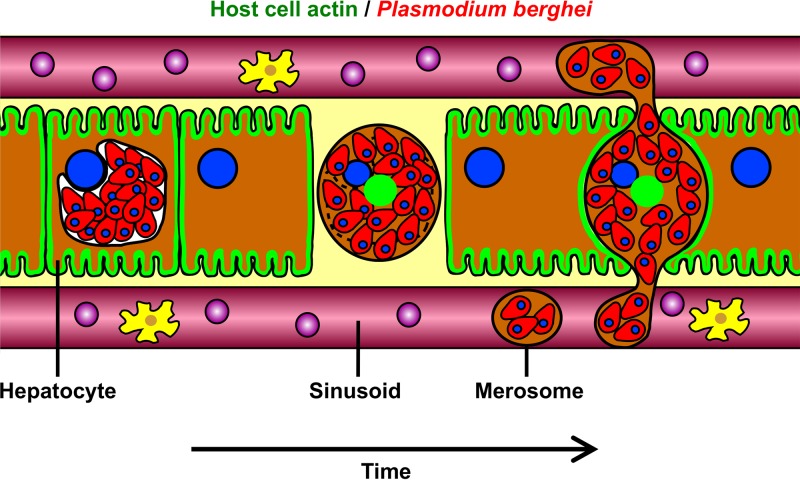
Model of *in vivo* release of merosomes. Upon rupture of the PVM, *Plasmodium* liver stage parasites induce dissociation of the actin cytoskeleton from the HCM, which leads to HCM destabilization. At the same time, the infected hepatocyte detaches from neighboring cells. The neighboring cells sense the detachment and close the tissue gap. Thereby, physical pressure is exerted on the destabilized infected cell, leading to release of merosomes into liver sinusoids, where merosomes bud off from the mother cell because of shear forces in the bloodstream.

In this study, we have shown that *Plasmodium* parasites induce the disconnection of the actin cytoskeleton from the HCM during their egress from host hepatocytes. In doing so, parasites appear to considerably destabilize the plasma membrane, which might be one of the key factors leading to merosome formation. *In vivo* release of merosomes might thereby be supported by the surrounding cells, which sense the detachment of the infected cell and close the tissue gap. This presumably exerts physical pressure on the destabilized infected cell, which leads to release of merosomes into liver sinusoids (Fig. 6). In support of this hypothesis, we saw rapid closure of the tissue gap induced by the detachment of infected cells when we analyzed liver stage egress in nearly confluent tissue-like primary hepatocyte cultures (see Movie S9).

10.1128/mBio.00139-17.9 MOVIE S9  *Plasmodium*-induced cell detachment in nearly confluent primary hepatocyte cultures. Primary hepatocytes derived from a Lifeact mouse (green) were infected with mCherry-expressing parasites (red). Parasite development was monitored by confocal live-cell time-lapse microscopy, and imaging was started at 56 hpi. The movie was acquired with a 10-min time interval between frames and is shown at four frames per second. Hours and minutes from the start of the movie are displayed. Results are representative of 30 analyzed egress events in two independent imaging experiments. Scale bar, 50 μm. Download MOVIE S9, AVI file, 2.4 MB. Copyright © 2017 Burda et al. 2017 Burda et al. This content is distributed under the terms of the Creative Commons Attribution 4.0 International license.

Under normal circumstances, actin is linked to membrane proteins by adaptor proteins such as ezrin, radixin, or moesin and the activity of these adaptor proteins depends on the phosphoinositide PIP_2_ in the plasma membrane (reviewed in reference 6). To analyze whether the phospholipid composition of the HCM is modulated during liver stage egress, we expressed fluorescent reporters for PIP_2_, PIP_3_, and PS in *P. berghei*-infected host cells. Interestingly, we observed the loss of all of these reporters from the HCM upon PVM rupture, suggesting that the respective host cell phospholipids are indeed modulated during liver stage release. Similarly, we saw that a GFP fusion protein of the PIP_2_-dependent actin-plasma membrane linker ezrin was rapidly lost from the HCM upon PVM rupture. It is therefore tempting to speculate that possible PIP_2_ loss from the HCM contributes to the dissociation of the actin cytoskeleton from the plasma membrane, although this needs to be proven experimentally.

In addition to the potential manipulation of host cell phospholipids during liver stage egress, we observed that the HCM is cleared of membrane proteins. Previously, it was shown that membrane proteins containing one transmembrane domain are rapidly lost from the HCM during liver stage egress (10). We now show that this holds true for proteins containing seven-transmembrane domains and for proteins that are bound to the plasma membrane by palmitoylation and myristoylation or by the addition of a GPI anchor. Importantly, the loss of membrane proteins also appears to occur *in vivo* during merosome formation, since the merosome membrane was shown to be devoid of the hepatocyte-specific single-pass transmembrane domain-containing protein ASGR1 (22). The loss of membrane proteins might be one explanation for why infected cells detach from the surrounding cells during merosome formation, as we could also show that the cell adhesion protein E-cadherin is lost from the HCM upon PVM rupture. Furthermore, the clearance of the HCM from membrane proteins might contribute to the observed loss of actin-plasma membrane linkage, because membrane proteins can serve as anchor sites in the HCM for the cytoskeleton.

The molecular mechanism of these pronounced manipulations of the HCM during *Plasmodium* liver stage egress is currently unclear. Given the fact that all of the alterations of the HCM only occurred after PVM rupture, we hypothesize that they are induced by one or several parasitophorous vacuole/PVM-localized parasite effector proteins that are released into the host cell upon PVM rupture. Interestingly, host cell PIP_2_ is also modulated by pathogenic bacteria. *Shigella flexneri* and *Vibrio parahaemolyticus* both use inositol phosphate phosphatases to hydrolyze PIP_2_ in the HCM. This decreases the force that tethers the plasma membrane to PIP_2_-binding cytoskeletal anchoring proteins and induces massive cell blebbing, which facilitates bacterial uptake or contributes to host cell lysis by interrupting plasma membrane integrity (reviewed in reference 23). It now remains to be shown whether *Plasmodium* liver stage parasites similarly express and release PIP_2_-modifying enzymes into the host cell cytoplasm upon PVM rupture. Alternatively, during egress, *Plasmodium* liver stage parasites could induce a signaling cascade in hepatocytes that ultimately results in the activation of host cell proteins that orchestrate the manipulation of the HCM and the formation of merosomes. Interestingly, a Gα(q)-coupled host signaling cascade was identified in *P. falciparum*-infected erythrocytes and *Toxoplasma gondii*-infected cells that was required for the cytolysis of infected cells and the release of parasites (24). Antibody-mediated depletion and knockdown of Gα(q) led to a severe defect in the egress of *P. falciparum* from erythrocytes and *T. gondii* from host cells. In contrast, egress of liver stage parasites appears to be unaffected in HeLa cell lines that lack Gα(q), while an egress defect for these cells infected with *T. gondii* is observed (Paul-Christian Burda, unpublished observation). This argues that differences in host cell signaling must exist between the lytic egress strategies of *T. gondii* and *Plasmodium* blood stages on one hand and the nonlytic merosome formation of *Plasmodium* liver stage parasites on the other hand.

Although *Plasmodium* blood and liver stages apparently differ in some aspects of egress, there are also some striking similarities. A dynamic pre-egress behavior of the infected RBC was shown to occur before merozoite release (25) that quite resembles the *Plasmodium*-induced blebbing of the HCM during the egress of liver stages described in this study. Furthermore, it has been demonstrated by high-speed video microscopy that egress of *Plasmodium* parasites from RBCs is the result of an elastic instability of the infected erythrocyte membrane (26). It was shown that after osmotic swelling of the infected erythrocyte, a pore opens and one or two merozoites are ejected. This is followed by outward curling and fast eversion of the erythrocyte membrane, whereby parasites are pushed forward. However, what induces the elastic instability of the erythrocyte membrane necessary for blood stage release is not completely understood so far, but disconnection of the lipid membrane from the underlying RBC cytoskeleton has been proposed as a potential explanation (26). It will therefore be highly interesting to investigate whether a similar pronounced manipulation of the host cell cytoskeleton and plasma membrane, which we describe in this study to occur during the egress of *Plasmodium* liver stages, might also happen during the release of blood stage parasites. In line with this, a recent study provided evidence that in the final minutes of blood stage egress, the RBC membrane abruptly loses its structural rigidity and collapses around parasites (5), suggesting that breakdown of the host cell cytoskeleton occurs during blood stage release as well.

In conclusion, this study identifies the disconnection of the HCM from the actin cytoskeleton and a potential manipulation of its phospholipid and protein content as a novel host cell modification triggered by *Plasmodium* liver stage parasites during egress. It thus provides a first insight into the molecular events that result in the formation of merosomes and thereby explains how hepatocyte-derived merozoites transit so successfully from the liver to the blood circulation.

## MATERIALS AND METHODS

### Ethics statement.

All of the experiments described here were conducted in strict accordance with the guidelines of the Swiss Tierschutzgesetz (animal rights law) and approved by the ethical committee of the University of Bern (permit BE109/13).

### Experimental animals.

The mice used in these experiments were between 6 and 10 weeks of age. BALB/c mice were from Harlan Laboratories or bred in the central animal facility of the University of Bern. Lifeact mice were obtained from the Theodor Kocher Institute of the University of Bern. Mosquito feeds were performed with mice anesthetized with ketamine (Ketavet) and medetomidine (Domitor) (Pfizer), and all efforts were made to minimize suffering.

### Parasite lines.

*P. berghei* ANKA parasites were used that either constitutively express GFP or mCherry in the parasite cytosol (27, 28) or that express an ExpI-mCherry fusion protein under the control of the liver stage-specific *lisp2* promoter (10).

### Cell cultivation, transfection, and infection.

Primary mouse hepatocytes were isolated and cultivated as described previously (29). HeLa cells (gift from Robert Menard, Pasteur Institute, Paris, France) were authenticated by short tandem repeat DNA profiling (Microsynth) and cultured as described before (30). We transfected 2 × 10^6^ HeLa cells with 4 µg of plasmid DNA of the following fluorescent reporter constructs with program T-28 in an Amaxa Nucleofector (Lonza): mCherry-utrophin (7) (Addgene plasmid 26740), GFP-utrophin (7) (Addgene plasmid 26737), GFP-C1-PLCdelta-PH (12) (Addgene plasmid 21179), AKT-PH-GFP (13) (Addgene plasmid 18836), Lact-C2-GFP (14) (Addgene plasmid 22853), ezrin-GFP (15), ezrin(T567D)-GFP (16) (Addgene plasmid 20681), E-cadherin–GFP (17) (Addgene plasmid 28009), A2BR-YFP (18) (Addgene plasmid 37202), GFP-DRD2 (19) (Addgene plasmid 24099), PM-GFP (20) (Addgene plasmid 21213), and GFP-GPI (TRAIL and DAF) (21). Cells were subsequently used to seed glass bottom dishes (MatTek, In Vitro Scientific) and infected the next day with *P. berghei* sporozoites as previously described (28).

### Live-cell time-lapse microscopy.

Late liver stage development and detachment of infected host cells were analyzed by live-cell time-lapse imaging. For this, either an LSM 5 Duo microscope (live mode, confocal line scanning) or an LSM 880 microscope (airy scan mode), each with a Zeiss Plan-Apochromat 63×/1.4 oil objective, was used for confocal microscopy. Alternatively, a Leica DM12000B with a Leica Plan-Apochromat 63×/1.2 water immersion objective was used for widefield microscopy. For multiposition time-lapse imaging, the Zeiss LSM Multitime-Macro, the ZEN 2.1 software, or the Leica Application suite 2.6 was used. During imaging, cells were kept in a CO_2_ incubator at 37°C. Image processing was performed with ImageJ.

### Analysis of actin localization in detached cells

We seeded 5 × 10^4^ HeLa cells or 1 × 10^5^ primary hepatocytes derived from Lifeact mice per well of 24-well plates and infected them with sporozoites the next day. To analyze the influence of PVM rupture on actin morphology, 10 µg/ml E64 (Sigma) was added to parasites between 55 and 56 h postinfection (hpi). Detached cells were harvested at 65 hpi. One microgram of Hoechst 33342 (Sigma) per milliliter was added to detached cells of primary Lifeact hepatocytes, which were observed live by epifluorescence microscopy. For phalloidin staining of detached HeLa cells, first the cell culture supernatant containing detached cells was centrifuged and then the supernatant was carefully removed. Detached cells were then fixed in 100 µl of 4% paraformaldehyde–phosphate-buffered saline (PBS) for 10 min. They were washed once in 500 µl of PBS and then permeabilized and stained for 10 min in 100 µl of 10% fetal calf serum–PBS containing 0.1% TX-100, phalloidin-Alexa Fluor 488 (diluted 1:250; Molecular Probes), and 1 µg/ml 4',6-diamidino-2-phenylindole (DAPI; Sigma). After washing with 500 µl of PBS, stained detached cells were immediately observed by epifluorescence microcopy. All centrifugation steps were performed for 5 min at 200 × *g*.

### Statistical analysis.

For statistical analysis of differences between two groups, we used an unpaired two-tailed *t* test, and for statistical analysis of differences among more than two groups, a one-way analysis of variance (ANOVA), followed by a Holm-Sidak multiple-comparison test, was performed. *P* values of <0.05 were considered significant.
